# Addition of Lymphatic Stimulating Self-Care Practices Reduces Acute Attacks among People Affected by Moderate and Severe Lower-Limb Lymphedema in Ethiopia, a Cluster Randomized Controlled Trial

**DOI:** 10.3390/jcm9124077

**Published:** 2020-12-17

**Authors:** Jan Douglass, Fikre Hailekiros, Sarah Martindale, Hayley Mableson, Fikre Seife, Tesfahun Bishaw, Mekdes Nigussie, Kadu Meribo, Mossie Tamiru, Getnet Agidew, Susan Kim, Hannah Betts, Mark Taylor, Louise Kelly-Hope

**Affiliations:** 1 Centre for Neglected Tropical Diseases, Department of Tropical Disease Biology, Liverpool School of Tropical Medicine, Liverpool L3 5QA, UK; sarah.martindale@lstmed.ac.uk (S.M.); hayley.mableson@liverpool.ac.uk (H.M.); hannah.betts@lstmed.ac.uk (H.B.); mark.taylor@lstmed.ac.uk (M.T.); Louise.Kelly-Hope@lstmed.ac.uk (L.K.-H.); 2 National Podoconiosis Action Network, Addis Ababa 1000, Ethiopia; fikre_h@napanethiopia.org; 3 Institute of Infection, Veterinary and Ecological Sciences, University of Liverpool, Liverpool L69 3BX, UK; 4 Diseases Prevention and Control Directorate, Federal Ministry of Health, Addis Ababa 1000, Ethiopia; fikreseife5@gmail.com (F.S.); bishawtesfahun@gmail.com (T.B.); mekdesnigussie880@gmail.com (M.N.); meribokadu@gmail.com (K.M.); mossietamiru2015@gmail.com (M.T.); gagidew@iocc.org (G.A.); 5 College of Medicine and Public Health, Flinders University, Bedford Park, SA 5042, Australia; sus97nz@gmail.com

**Keywords:** lymphedema, lower limb, filariasis, podoconiosis, self-care, breathing, exercise, skin mobilisation, lymphatic massage

## Abstract

Lymphedema causes disability and exacerbates poverty in many countries. The management of lymphatic filariasis (LF) and podoconiosis-related lymphedema involves daily hygiene to reduce secondary infections, but self-massage and deep-breathing, which have proven beneficial in cancer-related lymphedema, are not included. A cluster randomized trial in northern Ethiopia investigated the effects of lymphatic stimulation for people affected by moderate to severe lymphedema. Participants were allocated to either standard (control *n* = 59) or enhanced (intervention *n* = 67) self-care groups. Primary outcomes were lymphedema stage, mid-calf circumference, and tissue compressibility. Secondary outcomes were the frequency and duration of acute attacks. After 24 weeks, fewer patients were assessed as severe (control −37.8%, intervention −42.4%, *p* = 0.15) and there were clinically relevant changes in mid-calf tissue compressibility but not circumference. There was a significant between-group difference in patients who reported any acute attacks over the study period (control *n* = 22 (38%), intervention *n* = 7 (12%), *p* = 0.014). Daily lymphedema self-care resulted in meaningful benefits for all participants with a greater reduction in acute episodes among people performing lymphatic stimulation. Observations of a change in lymphedema status support earlier findings in Bangladesh and extend the demonstrated benefits of enhanced self-care to people affected by podoconiosis.

## 1. Introduction

Lymphatic disorders are a major cause of disability worldwide, and the extreme suffering of people affected by lymphatic filariasis (LF)-related lymphedema and hydrocele was the impetus for establishing the Global Programme to Eliminate Lymphatic Filariasis (GPELF) [1]. The World Health Organization (WHO) provides guidelines for LF endemic countries to carry out the morbidity management and disability prevention (MMDP) aspects of the GPELF [2], and recommend the same guidelines be used to support people affected by podoconiosis-related lymphedema [3]. LF and podoconiosis are co-endemic in 20 countries [4] and in both cases the burden of disability occurs disproportionately among the poorest populations [5,6] and the most severe cases require considerable family and community support [7]. The GPELF has been successful in stopping LF transmission in 17 of 72 endemic countries [8], so there should be no new cases of LF-related lymphedema in those countries. New cases of podoconiosis can also be prevented when subsistence farmers in endemic areas are able to wear closed shoes and live in houses with sealed flooring [9]. However, people who already have clinically manifest lymphedema face decades of disease progression, stigma, and social exclusion [10,11,12].

Lifelong and not life-threatening, lymphedema is ideally managed within a community-based home-care model. Daily hygiene practices are the central component of long-term self-care and reduce the risk of acute and disabling secondary infections, frequently referred to as acute attacks. People affected by advanced lymphedema who follow this meticulous limb washing and drying regime will benefit greatly from a reduction in illness and impairment, but may notice less reduction in limb size than can be seen among people affected by more mild lymphedema who follow the same recommendations [13]. Current MMDP guidelines for lymphedema self-care include only calf-pump exercises (rising up and down on the toes) to support lymph flow, but research on other lymphedema populations suggests that clearing proximal lymph pathways through diaphragmatic breathing and proximal muscle pump exercises [14,15,16,17], and more directly stimulating the lymphatic system through massage [18], may improve outcomes and even prevent lymphedema from forming in those at risk [19,20]. Few lymphatic stimulating practices are offered to people affected by LF- and podoconiosis-related lymphedema but could be added to current recommendations without additional financial burden [19], and may increase the benefits that people derive from adherence to their daily self-care efforts [21,22].

Lymphedema is a progressive skin and subcutaneous tissue disease resulting from lymphatic insufficiency and failure [10]. When lymph removal is inhibited, complex connective tissue disease ensues with a high level of variability between individuals [23]. Chronic inflammation leads to structural tissue changes, including fibrosis and hyper adiposity of subcutaneous tissue, and disfiguring skin pathologies such as dermal sclerosis and papillomatosis. Wound healing is inhibited and fungal overgrowth contributes to intertrigo (macerated skin between the toes) [24]. Since lymphedema usually progresses slowly after a long period of latency, sub-clinical connective tissue changes will precede more overt signs. These covert changes can be detected by several methods including tissue tonometry [25,26] which quantifies tissue compressibility or stiffness and has been used to detect sub-clinical changes among asymptomatic young people in LF endemic regions in Papua New Guinea and Myanmar [25,27]. Early detection of clinically relevant tissue change allows for meaningful analysis of research outcomes without waiting for overt clinical changes to manifest, supports early and preventative intervention, and facilitates provision of timely evidence-based information to guide health service delivery with an efficient use of limited resources.

A cluster randomised trial was conducted in Bangladesh and Ethiopia to determine if the addition of lymphatic stimulating activities, including diaphragmatic breathing to clear the central lymphatics; thigh exercises to support emptying of the proximal thigh vessels; lymphatic massage to encourage fluid movement out of congested tissues, and skin mobilisation of affected limbs could benefit people affected by moderate and severe lower-limb lymphedema [28]. Both groups were trained in the currently recommended standard self-care activities [29], and the intervention group were also trained in the added lymphatic stimulating enhanced self-care practices [28]. These countries were chosen for their differing populations and stage of MMDP activities. Bangladesh is endemic for LF only and the LF MMDP program has been rolled out to all endemic regions at least once [30]. In Ethiopia, the national MMDP program is not yet scaled at national levels, and podoconiosis and LF are co-endemic in 29 of 70 LF endemic districts [31]. The aim of this paper is to present the results on the Ethiopia arm of the study, highlighting the differences between LF- and podoconiosis-related lymphedema, and reinforcing the economic argument for increased efforts to deliver quality MMDP services to affected populations.

## 2. Materials and Methods

### 2.1. Study Design, Research Personnel and Data Collection

**Figure 1:**
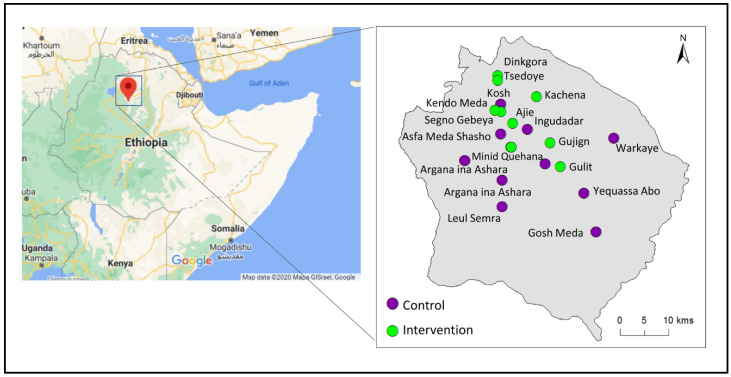
Map of all included HP in Simada District by allocation group.

Simada District in north western Ethiopia has a high prevalence of LF-related lymphedema and is co-endemic for podoconiosis [32]. Cluster randomization was used to allocate all accessible health posts (HP) in Simada District (*n* = 20) to either the standard self-care (control) [2], or enhanced self-care (intervention) [28] groups. All participants enrolled in each HP were trained in the same self-care protocol with follow-up measures and questionnaires administered at 4-, 12-, and 24-weeks. Figure 1 is a map of all included HP in Simada District by allocation group.

Two research teams were formed from local Federal Ministry of Health (FMOH) personnel in Simada, and program managers from the FMOH LF elimination program and the National Podoconiosis Action Network (NAPan), which is a non-governmental implementing partner for MMDP delivery in Ethiopia. Each research team was composed of a clinical officer, a data collector, a lymphedema self-care trainer, and a team supervisor. The study was conducted in accordance with the Declaration of Helsinki [29], approved by the Amhara Public Health Institute Research Ethics Review Committee and the Liverpool School of Tropical Medicine Research Ethics Committee (approval no. 012-18), and registered on the ISRCTN Registry (trial number 16,764,792). All subjects gave their informed consent for inclusion before they participated in the study.

At each HP, a maximum of 10 adults (age ≥ 18 years) affected by lower-limb lymphedema from any cause, which was assessed as stage 3 or higher according to Dreyer, et al. [33] were invited to participate in the study. A designated adult caregiver for each patient, and two health extension workers (HEW) were also enrolled and trained in the allocated lymphedema self-care protocol. Information about the study was explained by the trainers and supervisors in Amharic, and every participant (patient, caregiver, and HEW) was given a written information sheet (in Amharic) to keep. Volunteers were assessed by the clinical officer to ensure they had lymphedema of at least stage 3 [26]. Patients (and their designated caregiver) were excluded if the patient had a diagnosis of co-morbidities known to contribute to edema such as diabetes or congestive heart failure. Limiting patient enrolments to no more than ten at each HP avoided over-loading the HEWs who were asked to provide ongoing support. However, all volunteers and caregivers, including those who were not enrolled in the study, were invited to participate in the lymphedema self-care training.

### 2.2. Outcome Measures and Data Collection

Detailed data collection methods have been published [21,28,34]. The primary outcome was change in lymphedema status as determined by a combination of; (1) lymphedema stage [33], (2) mid-calf circumference, and (3) mid-calf tissue compressibility quantified by tissue tonometry using the Indurometer [35]. Secondary outcome measures were (1) frequency and duration of acute attacks, (2) days of work lost due to lymphedema, and (3) number of interdigital lesions and entry lesions (unhealed wounds) on the affected leg(s). The clinical officer assessed each leg and if there were any clinical sign of lymphedema the leg was classified as stage 1–7 according to indicative features [33]. Legs without any sign of lymphedema were classified as stage 0. Circumference and tissue compressibility measures were taken at the mid-point of the calf. A retractable tape measure in centimetres (cm) was used to locate the mid-point between the popliteal crease and the base of the heel of each leg with the patient lying prone, and to measure the mid-calf circumference. The Indurometer (BME-1563; Flinders and SA Biomedical Engineering, Bedford Park, South Australia) was used to quantify tissue compressibility at the same mid-calf point. A detailed description of the Indurometer and its operation were published in an intra-rater reliability analysis on the study population [34].

Socio-demographic information on patients and caregivers was collected by questionnaire and patients were also asked about their medical status and lymphedema history. The questionnaire also elicited the history of acute attacks and working days lost due to lymphedema over the previous one- and six-months [36]. The clinical officer administered the questionnaires, determined the stage of lymphedema, collected the circumference and Indurometer measures and counted the interdigital and entry lesions present. Participant responses and device scores were entered by the data collector to the Open Data Kit Collect (ODK Collect) application [28] which had been loaded to an electronic tablet (Samsung Galaxy Tab A 10.1). Data were collected early in the day and not later than 2 pm to minimise the effect of any diurnal fluctuation in swelling [10,37].

## 3. Intervention

A detailed description of the standard and enhanced self-care protocols has been published [28]. The standard self-care group (control) were trained in the currently recommended daily practices of thoroughly washing and drying any affected body parts. Antifungal creams were applied to interdigital lesions, and other entry lesions were covered with Vaseline. Ankle range of motion and calf pump exercises were demonstrated, and information was provided on managing acute attacks. Each patient was given a large plastic bowl for washing, enough soap, wash cloths and topical creams to complete the study, and advised to attend the HP if additional medication was required during acute attacks. The intervention group were trained in the same self-care protocol as the control group with the addition of self-lymphatic massage, skin mobilisation, deep breathing and thigh muscle exercises, recommendations to eat at least one serving of fresh fruit or vegetables on four or more days per week, and to drink at least five glasses of water each day. Adherence to the self-care protocol and the amount of time given by caregivers were recorded in a daily journal. A paper journal for each 4-week period and a pencil were provided and patients and caregivers were instructed in how to enter tick marks to a daily activities chart. At each follow-up, the completed journal(s) were collected and checked, participants were reminded of the daily self-care activities, and enough journals for the next period were provided.

## 4. Analysis

For analysis purposes, patients were stratified by the stage of their worst affected leg to either moderate (stages 3 and 4) or severe (stages 5–7). Individual legs without apparent edema were stratified as stage 0, and legs at stages 1 and 2 were classified as mild [17]. A subgroup of legs with mossy lesions at the feet but no observable swelling was classified as Stage ‘P’ based on notes provided by the data collectors and on review of patient photographs.

Data on lymphedema stage, mid-calf circumference and Indurometer scores, and number of interdigital and entry lesions were analyzed by each leg, with the stage of each leg at baseline included as a fixed effect for circumference and Indurometer scores. The stage of the most severe leg at baseline was used to stratify whole person variables such as frequency and duration of acute attacks, and the number of working days lost due to lymphedema which were analyzed using Poisson logistic mixed effects models. Time point, group (control or intervention), and maximum stage (stage P, moderate, or severe) at baseline were included in the model as fixed effects. Random effects were used for cluster and patient.

A change of either ≥10% or ≥2 cm in circumference was considered clinically relevant, and a change of ≥10% was used for Indurometer scores [30,31]. All analyses were performed using Stata 15.1 (StataCorp, College Station, TX, USA) and a *p*-value of less than 0.05 was considered statistically significant. Descriptive statistics are presented as median with interquartile range for continuous variables and as frequency with percentage for categorical variables. Linear mixed effects models were used to analyze Indurometer and circumference measurements for each leg using the identity number as a random effect to account for non-independence of the leg pairs belonging to one individual. Time point and group (intervention or control) were included in the model as a priori. Leg dominancy and stage (none, mild, moderate or severe) at baseline were tested for inclusion in the model as fixed effects. All interactions were examined for inclusion. Random effects were used for cluster, patient, and leg (right or left) for Indurometer and circumference. Data from the first week of the first journal (week one) and the first week of the last journal (week 21) were extracted to determine adherence to the allocated self-care regimen.

## 5. Results

### 5.1. Participants

**Figure 2:**
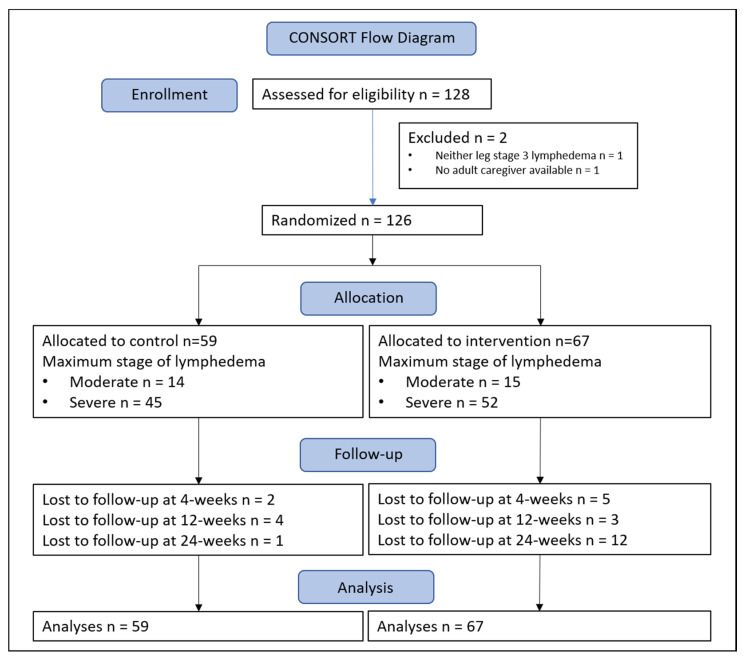
Flow of patients through the study.

A total of 128 people affected by lower limb lymphedema and 127 adult caregivers were enrolled. One patient (and their caregiver) was excluded as neither leg had progressed to stage 3, and another was excluded as they did not have an adult caregiver. At the 4- and 12-week follow-ups, retention was 94%, and 90% of patients returned for final 24-week measures (*n* = 113). A flow chart of all patients through the study can be seen in Figure 2.

Most patients were in their fifth decade (median 57.5 years, IQR 50, 66), just over half were male (*n* = 70, 55.6%) and three-quarters had severe lymphedema in at least one leg (*n* = 97, 77%). The majority had bilateral lower-limb lymphedema (*n* = 102), and 19% had unilateral lymphedema (right leg *n* = 16, left leg *n* = 8). One person with bilateral affected legs also had one arm affected. There were 18 people with their most affected leg initially classified as stage 6 due to the presence of skin pathologies [33], but as they had no observable swelling they were reclassified to stage P (14.3%). At baseline, 92% (*n* = 116) of patients reported at least one acute attack in the previous six-months (median episodes, 3 (IQR 1, 4) range 0–35), and 60% (*n* = 73) reported one or more episodes in the previous one-month (median 1, IQR 0, 1, range 0–3). Similarly, 79% (*n* = 99) of patients had lost one or more working days due to lymphedema in the six-months before the study (median 9.5 days IQR 3, 18 range 0–180 days), and 58% (*n* = 73) reported being unable to work on at least one day in the previous one-month (median 2 days IQR 0, 4 range 0–30 days).

Fifty-nine patients and their caregivers were allocated to the standard self-care group (control), and 67 were allocated to the enhanced self-care group (intervention). There were no significant between-group differences for maximum stage of lymphedema, mid-calf circumference, frequency of acute attacks, days of work lost due to lymphedema, or number of interdigital and entry lesions. There were significant between-group differences in the use of appropriate footwear with twice as many patients in the intervention group wearing closed shoes at baseline than controls (*p* = 0.004), and in mean Indurometer score (6.9%, *p* = 0.011) which was not clinically relevant. When stratified for stage, the between-group difference was found only among severe cases and did not reach clinical significance (9.3%, *p* = 0.014). Patient characteristics at baseline are given in Table 1.

Data were available on 252 legs and 60% were affected by severe lymphedema (control *n* = 71, 60%, intervention *n* = 80, 60%, *p* = 0.79) and 23% were moderate (controls *n* = 25, 21.2%, intervention *n* = 32, 23.9%). The remaining 17% were either mild (control *n* = 7, 6%, intervention *n* = 8, 6%) or had no clinical sign of lymphedema (stage 0, control *n* = 15, 13%, intervention *n* = 14, 10%). Mossy lesions in the absence of visible swelling were present on 36 legs (14%) and these were reclassified to Stage P (control *n* = 14 12%, intervention *n* = 22, 16%). Lymphedema stage by group and time are given in Supplementary Table S1.

Adherence to the allocated self-care protocol was high throughout the study period without any between-group differences. One week after the training session all participants recorded washing and drying their legs at least once per day with most performing this activity twice or more every day (control *n* = 49, 83%, intervention *n* = 60, 94%). At 21-weeks the proportion of patients who were performing the hygiene routine twice or more each day had reduced slightly (control *n* = 47, 78%, intervention *n* = 53, 83%) and one person did not record daily leg washing that week. Adherence to the additional enhanced care activities by the intervention group at week one was 89% with the thigh exercises performed the least (83%) and self-massage performed the most (98%). At week 21, overall adherence was 77% with breathing exercise performed the least (69%) and skin mobilisation performed the most (81%). During week one, 91% (*n* = 61) of patients in the intervention group reported eating the required portions of fresh fruit and vegetables, and 63% (*n* = 35) reported consuming these at 21-weeks. Water consumption in the intervention group was also high with 100% of patients reporting at least five glasses per day at week one, and 73% (*n* = 40) at week 21. At 24-weeks, the use of appropriate footwear had increased to 64% of all participants (control *n* = 31 (53%), intervention *n* = 41 (75%), *p* = 0.041) with the greatest improvement in the control group (19% vs. 13%).

During week one, caregivers had assisted patients with lymphedema self-care for up to 4-days in both groups (mean cumulative hours control 7.12 ± 5.8, intervention 5.89 ± 5.59). At week 21, caregiver time had remained consistent in the control group (6.98 ± 6.21), but almost halved in the intervention group (3.95 ± 5.24, *p* = 0.006).

### 5.2. Primary Outcomes

#### 5.2.1. Change in Lymphedema Stage

**Figure 3:**
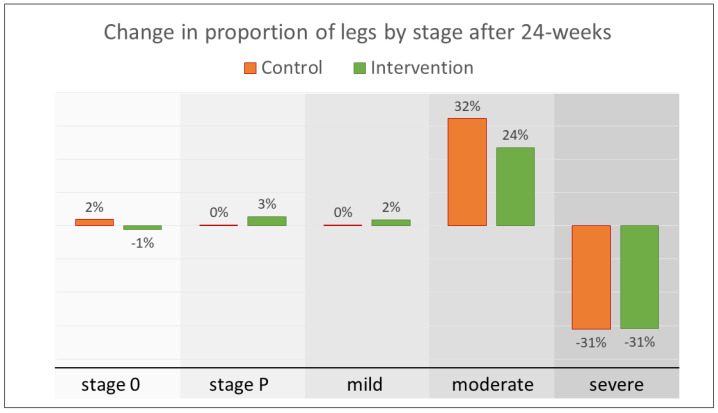
Change in proportion of legs by group and stage.

At 24-weeks there had been a 21% reduction in the proportion of people assessed as having severe lymphedema in at least one leg (control −37.8% of legs, intervention −42.4% of legs, *p* = 0.15). At 12-weeks there was a significant between-group difference in the proportion of legs at each stage (*p* = 0.013) driven by fluctuations between mild, moderate, stage P, and stage 0, whereas severe cases changed slowly and steadily over the study period. There were no between-group differences in the proportion of legs at each stage at 24-weeks (*p* = 0.36). Figure 3 shows the change in the proportion of legs at each stage after 24-weeks of self-care, and the number of legs at each stage by group and time are given in Supplementary Table S1.

#### 5.2.2. Mid-Calf Circumference

**Figure 4:**
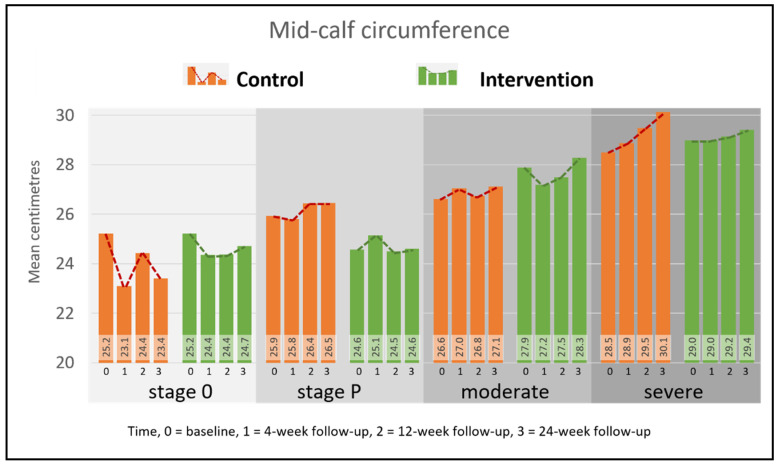
Change in mid-calf circumference by group and stage.

The mean circumference of all legs at baseline was 27.35 cm (± 3.71) with no significant between-group differences (control 27.28 cm ±3.46, intervention 26.50 cm ±3.93, *p* = 0.78). Legs affected by severe lymphedema were larger at mid-calf than legs assessed at stage 0 (mean difference 3.4 cm, control severe 28.51 cm ± 3.79 vs. stage 0 25.22 cm ± 3.56, intervention severe 28.82 cm ± 4.48 vs. stage 0 25.22 cm ± 1.56). The circumference of legs reclassified as stage P had a similar circumference to legs at stage 0 (stage P, control 25.94 cm ± 1.48, intervention 24.66 cm ± 2.34). Mid-calf circumference did not change significantly over the study period, however it is notable that there were fluctuations in both directions in stage 0, stage P and moderate cases, whereas severe cases showed a smaller but steady increase at each follow-up (Figure 4 and Supplementary Table S2).

#### 5.2.3. Midcalf Tissue Compressibility

**Figure 5:**
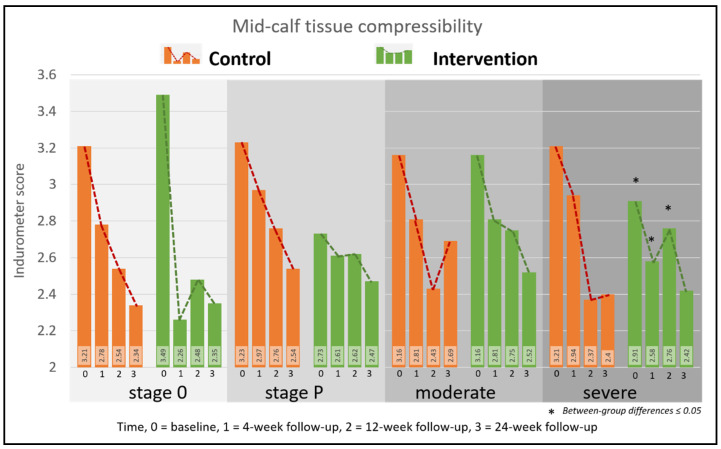
Change in Indurometer scores by group and stage.

The mean Indurometer score for all legs at baseline was 3.08 ± 0.71 and after 24-weeks there had been significant and clinically relevant reductions in both groups (control 21.2%, intervention 17.8%, *p* < 0.0001 for both). There were significant between-group differences at 4- and 12-weeks (*p* = 0.005, *p* = 0.049) but not at 24-weeks (*p* = 0.64). Figure 5 shows the change in tissue compressibility by stage and all Indurometer scores are given in Supplementary Table S3.

#### 5.2.4. Frequency of Acute Attacks Previous One- and Six-Months

**Figure 6:**
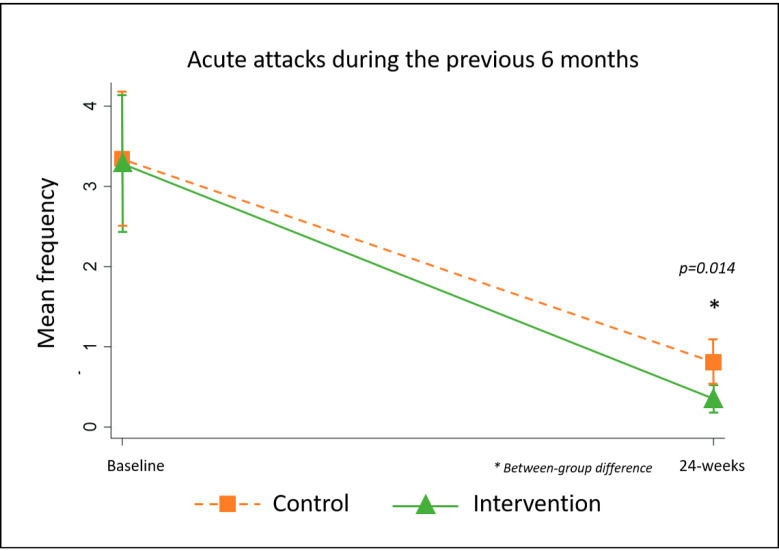
Frequency of acute attacks by group.

From baseline to 24-weeks, the proportion of patients who reported no acute attacks during the previous one-month more than doubled from 41% (*n* = 55) to 89% (*n* = 100). Similarly, while 90% of patients (*n* = 114) had experienced at least one acute attack during the six-months before the study, only 29 people (25%) reported any episodes during the study period with a significant between-group difference favouring the intervention (control *n* = 22, intervention *n* = 7, *p* = 0.014, Figure 6).

**Figure 7:**
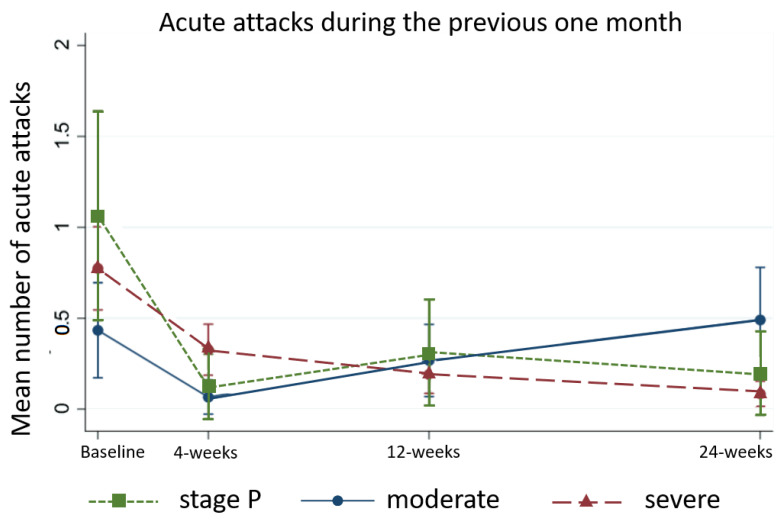
Frequency of acute attacks by stage.

People affected by moderate and stage P lymphedema had the biggest improvements at 4-weeks whereas severe cases continued to improve to 24-weeks (Figure 7).

#### 5.2.5. Duration of Acute Attacks and Days of Work Lost to Lymphedema

**Figure 8:**
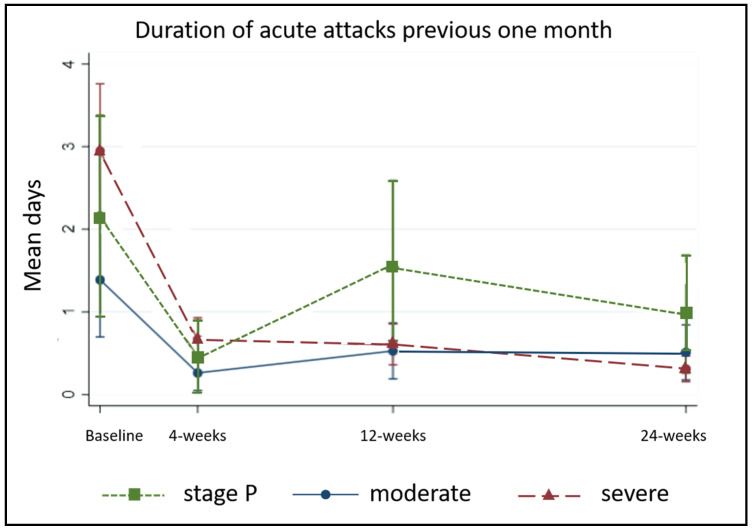
Duration of acute attacks by stage.

At baseline, patients reported acute attacks lasting up to 30 days over the previous one-month (median 1 day (1, 0) for both groups, *p* = 0.48) and 12 patients reported episodes lasting seven or more days. The most significant improvements were observed in the first four weeks, and at 24-weeks only two people reported episodes lasting more than one day. Severe cases were the most affected at baseline and achieved the greatest reduction over 24 weeks (Figure 8).

**Figure 9:**
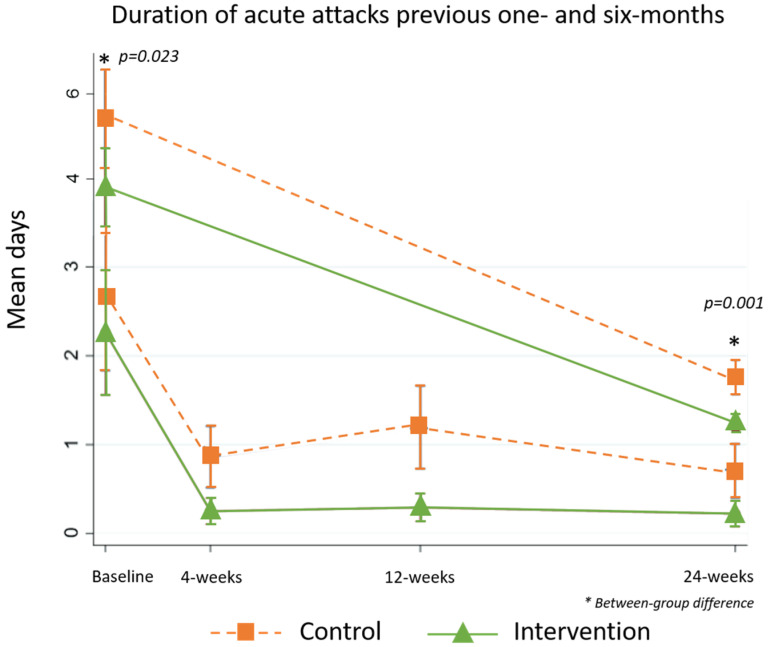
Duration of acute attacks by group.

There was a between-group difference in the duration of acute attacks over the previous six-months at baseline (control median 4 days (3, 5), intervention median 3 days (3, 4), *p* = 0.023), and both groups experienced significant improvement over the study period (control median 0 days (0, 3), intervention median 0 days (0, 0), *p* = 0.001, Figure 9).

**Figure 10:**
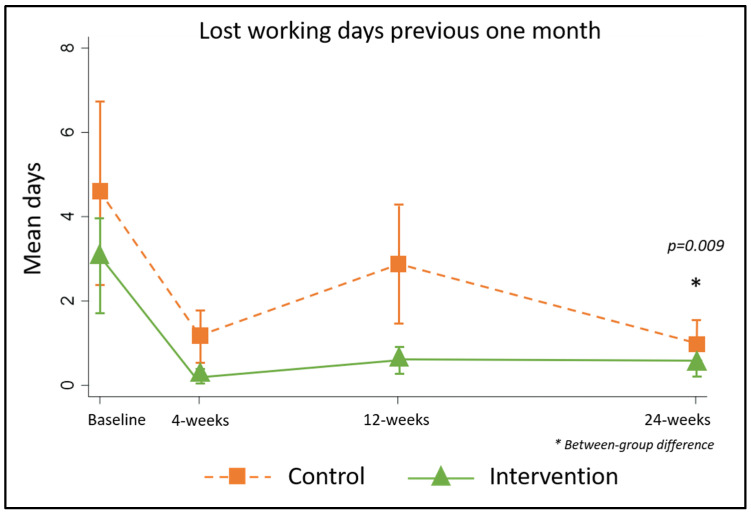
Number of lost working days by group.

At baseline, 58% of patients (*n* = 73) reported being unable to work due to lymphedema on at least one day during the previous month, and 12 people were unable to work on eight days or more (control median 3 (0, 6), intervention median 1.5 (0, 4), *p* = 0.22, range 0–30 days). At 24 weeks, only 11% of patients reported any working days lost in the previous one-month (control *n* = 10 (17%), intervention *n* = 3 (5%), *p* = 0.13) and only one person reported being unable to work on more than 8 days. The greatest improvements were achieved in the first four weeks and there were significant between-group differences favouring the intervention at 24-weeks (control median 0 (0, 5), intervention 0 (0, 0), *p* = 0.009, Figure 10).

**Figure 11:**
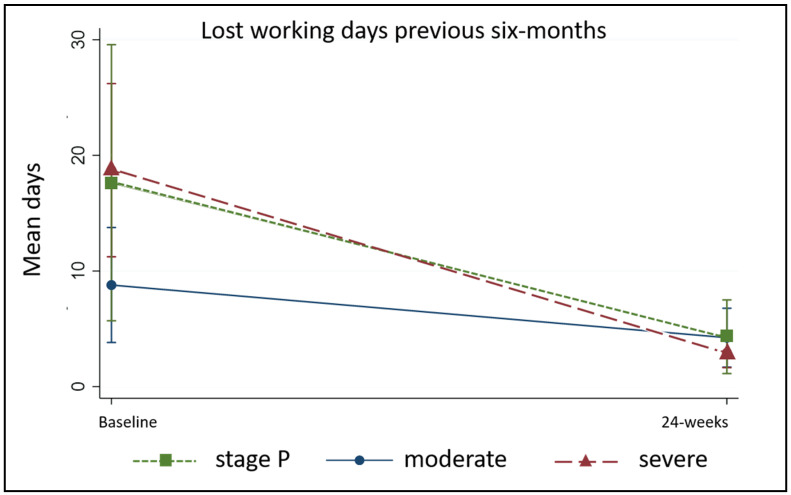
Number of lost working days by stage.

During the six months prior to the study, 79% (*n* = 99) of patients reported an average loss of 13 working days (± 18.06) with no between-group differences (control median 10 days (4, 20), intervention median 7 days (0, 15), *p* = 0.17, range 0–180 days). Of these, 40 people had been unbale to work on 15 days or more, but at 24-weeks only four people reported being unable to work on ≥15 days (range 0–60 days). Only 29 patients (26%) reported any working days lost during the study period and there were significant between-group differences favouring the intervention (control median 0 (0, 5), intervention 0 (0, 0), *p* = 0.009). People affected by severe and stage P lymphedema in both groups had been more affected at baseline and also had the greatest improvement at 24 weeks (Figure 11). Data on acute attacks and lost working days are given in Supplementary Table S4.

#### 5.2.6. Entry Lesions and Interdigital Lesions

At baseline, interdigital lesions were found between the toes on 41% of all legs (*n* = 103), and over half of these had three or more affected toe spaces (*n* = 64, 25% of all legs). After 24 weeks, only 11 legs had any interdigital lesions (5% of all legs) and eight legs had three or more affected toe spaces. Similarly, entry lesions were found on 22 legs (9%) at baseline and five legs had at least two lesions, but at 24-weeks there was a single lesion present on four legs only (2%).

## 6. Discussion

The introduction of daily self-care for people affected by LF- and podoconiosis-related lymphedema resulted in significant and meaningful benefits, including an improvement in lymphedema status, reduction in the frequency and duration of acute attacks and the number of days they were able to work, and fewer interdigital and entry lesions. Multiple studies have reported on the benefits of a daily hygiene routine for lymphedema [13,36,38,39,40], and this core component of self-care was included in the allocated protocol for both groups in our study [28]. In keeping with previous reports, we found larger changes in measures of lymphedema status occurring more readily among people affected by earlier stages [41,42], whereas lymphedema status changed more slowly among people affected by more advanced disease who benefited most from the reduction in acute attacks [13,36].

Primary outcome measures on lymphedema status in Ethiopia echoed the results reported on the Bangladesh arm of the study [21] which showed a greater change in connective tissue composition in the enhanced self-care group. Results on secondary outcomes in Ethiopia present further between-group differences favouring the intervention which were not observed in the Bangladesh cohort. In Ethiopia, people practicing the lymphatic stimulating activities had significantly fewer acute attacks which resolved more quickly, lost fewer working days than the standard self-care group and required less support from their caregivers than people performing the standard self-care protocol. When people affected by lymphedema are able to return to work or contribute to family resources, the economic gains are amplified by the reduction in demands on caregivers [7] with significant flow on benefits to the community [5,29,43].

Acute attacks and lost productivity are strongly correlated to secondary bacterial and fungal infections, poor skin integrity, and the presence of entry lesions, either interdigital skin maceration (intertrigo) or unhealed wounds [44]. Routine washing and drying of affected body parts removes aggravating agents such as soil, bacteria and fungus from the external skin, but microorganisms that have already crossed the skin barrier must be dealt with by the lymphatic and immune systems, which normally work hand in hand to clear this constant invasion. When lymphatic congestion occurs, invading agents accumulate in the tissues triggering chronic inflammatory responses, immune trafficking is reduced, wound healing is delayed, and a cycle of increasing infection against a background of deteriorating lymphatic function is established. The lymphatic stimulating activities in the enhanced self-care protocol support the removal of these accumulated agents, and dietary support for the immune system supports wound healing. In Bangladesh where water is plentiful and fresh fruit and vegetables are part of the daily diet, we did not find significant between-group differences in acute attacks. In Simada District, people must fetch and carry their daily water and most families cannot afford fresh fruits and vegetables on a regular basis. In this setting, the introduction of lymphatic simulation exercises and immune support may account for the greater improvement observed in the intervention group.

Lymphedema from LF and podoconiosis share many similarities, including irreversible swelling, limb deformity and skin pathologies, but there are fundamental differences. Whereas the lymphatic disruption in LF is proximal (worm nests in the groin or armpits), in podoconiosis the exposure is at the feet where irritant soils create a cascade of inflammatory reactions, and skin and connective tissue changes may precede lymphatic damage [3,9]. As the enhanced self-care protocol was designed to assist people affected by lymphedema from all causes, we made no attempt to differentiate the cause of lymphedema in our study. However, these etiological differences did present challenges in assigning LF stages to podoconiosis-related lymphedema [37,45]. The criteria described by Dreyer, et al. [33] assumes a linear pathogenesis of clinical signs arising from proximal interruption to lymph flow, but several stages of lymphedema may be present in a single limb as is illustrated by the reclassified stage P cases in Ethiopia [24,37]. In our study, mid-calf circumference and tissue compressibility fluctuated the most among moderate and stage 0 cases in both countries and stage P cases in Ethiopia. Although there was no observable swelling present on stage 0 and stage P legs, the notable changes in tissue compressibility at baseline were similar to those detected among young asymptomatic people infected with LF in Myanmar [27], and suggest a covert accumulation of extracellular fluid typical of latent lymphedema. Monitoring for very early and subclinical change is a key strategy in preventing cancer-related lymphedema [26] and investigation on early detection and intervention strategies among LF-and podoconiosis affected populations are also warranted.

There were limitations in study design and data collection methods specific to the Ethiopia arm of the study. The co-incidence of data collection and fasting days meant many people did not drink the cup of water that was offered before objective measures to control for hydration which is known to influence tissue compressibility [46], however this affected both groups equally. The interpretation of Indurometer scores may be subjective if not supported by clinically relevant changes in other objective parameters, and minimal literature is available to assist with clarification. Nevertheless, the inclusion of a measure to detect subclinical changes was also a strength of the study, and the similar patterns of tissue change found in both Ethiopia and Bangladesh support the interpretation made. The use of an LF-staging system necessitated some cases to be reclassified after data collection had ceased, however the absence of swelling was able to be visually confirmed by a review of photographs. The heterogeneous nature of lymphedema from differing causes underscores the need to establish effective personal practices that can become life-long daily habits.

## 7. Conclusions

This study emphasises the need for MMDP programmes to promote and support lymphedema self-care to all affected persons. As more countries achieve GPELF elimination targets in stopping LF-transmission and move to scale up MMDP programs to national levels, it is critical that the WHO provide guidelines for lymphedema self-care that are as up-to-date and evidence-based as possible. Results in the Ethiopia arm of this study support our previous recommendations to include lymphatic stimulating practices in lymphedema self-care for people affected by LF-and podoconiosis-related lymphedema.

## Figures and Tables

**Table 1:** Participant characteristics at baseline.

	Standard Self-Care (Control)	Enhanced Self-Care (Intervention)	*p* =
Participants	*n* = 59	*n* = 67	
Age, median (IQR)	58 (50, 65)	57 (50, 68)	0.77
Gender = female, *n* (%)	24 (41%)	32 (48%)	0.42
Marital status = married, *n* (%)	40 (68%)	46 (67%)	0.17
Literacy = illiterate, *n* (%)	44 (74%)	49 (73%)	0.56
Maximum stage of lymphedema =			
Moderate *n* (%)	14 (24%)	15 (22%)	0.86
Severe *n* (%)	45 (76%)	52 (78%)	
Severe cases reassigned as stage P *n* (%)	7 (12%)	11 (16%)	0.47
Interdigital lesions ≥ 1 lesion *n* (%)	54 (46%)	57 (43%)	0.68
Entry lesions ≥ 1 lesion *n* (%)	7 (6%)	15 (11%)	0.13
Acute attacks 1-month, median (IQR)	1 (0, 1)	1 (0, 1)	0.48
Lost workdays 1-month, median (IQR)	3 (0, 6)	1.5 (0, 4)	0.22
Acute attacks 6-months, median (IQR)	3 (1, 5)	3 (1, 4)	0.74
Lost workdays 6-months, median (IQR)	10 (4, 20)	7 (0, 15)	0.17
Appropriate footwear n (%)	20 (34%)	41 (62%)	0.004
Circumference, mean cm (SD)	27.3 (3.46)	27.4 (3.93)	0.78
Indurometer score, mean (SD)	3.20 (0.60)	2.98 (0.77)	0.011

Moderate lymphedema = stages 3–4, severe = stages 5–7, IQR = interquartile range, cm = centimetres, SD = standard deviation.

## Supplementary Materials

The following are available online at https://www.mdpi.com/2077-0383/9/12/4077/s1, Table S1: Lymphedema stage by group and time, Table S2: Mid-calf circumference in centimetres, Table S3: Mid-calf Indurometer score, mean (SD). Table S4: Frequency and duration of acute attacks and working days lost.
