# Supplementary material for: Addition of Lymphatic Stimulating Self-Care Practices Reduces Acute Attacks among People Affected by Moderate and Severe Lower-Limb Lymphedema in Ethiopia, a Cluster Randomized Controlled Trial
Source: J Clin Med. 2020 Dec 17;9(12):4077. doi: 10.3390/jcm9124077 (PMC7766500; doi:10.3390/jcm9124077)
Supplement: Supplementary file 1 [file jcm-09-04077-s001.pdf]

1. Supplementary Table 1: Lymphoedema stage by group and time

Time	Stage	Control (legs)	Intervention (legs)	p = [a]
Baseline		n = 118	n = 134	0.63
	0	12 (10.5%)	9 (6.7%)	
	1	5 (4.2%)	7 (5.2%)	
	2	2 (1.7%)	1 (0.7%)	
	3	23 (19.5%)	28 (20.9%)	
	4	2 (1.7%)	4 (3.0%)	
	5	0 (0.0%)	0 (0.0%)	
	6	60 (50.8%)	60 (44.8%)	
	7	0 (0.0%)	2 (1.5%)	
	P	14 (11.9%)	24 (17.9%)	
4-weeks		n = 114	n = 124	0.69
	0	5 (4.4%)	7 (5.5%)	
	1	5 (4.4%)	4 (3.1%)	
	2	0 (0.0%)	1 (0.8%)	
	3	42 (36.8%)	43 (33.6%)	
	4	0 (0.0%)	1 (0.8%)	
	5			
	6	48 (42.1%)	48 (37.5%)	
	7			
	P	14 (12.3%)	24 (18.8%)	
12-weeks		n = 112	n = 128	0.012
	0	5 (4.5%)	14 (10.8%)	
	1	8 (7.1%)	2 (1.5%)	
	2	8 (7.1%)	2 (1.5%)	
	3	38 (33.9%)	43 (33.1%)	
	4	0 (0.0%)	3 (2.3%)	
	5	0 (0.0%)	0 (0.0%)	
	6	39 (34.8%)	42 (32.3%)	
	7	0 (0.0%)	0 (0.0%)	
	P	14 (12.5%)	24 (18.5%)	
24-weeks		n = 116	n = 110	0.36
	0	10 (8.6%)	9 (7.8%)	
	1	6 (5.2%)	5 (4.3%)	
	2	1 (0.9%)	4 (3.4%)	
	3	62 (53.4%)	55 (47.4%)	
	4	0 (0.0%)	0 (0.0%)	
	5	0 (0.0%)	1 (0.9%)	
	6	23 (19.8%)	18 (15.5%)	
	7	0 (0.0%)	0 (0.0%)	
	P	14 (12.1%)	24 (20.7%)	

a) p-values calculated using Fisher's exact test

2. Supplementary Table 2: Mid-calf circumference in centimetres

Time		Stage 0	Stage P	Moderate	Severe
Baseline	Control	25.2	25.9	26.6	28.5
	Intervention	25.2	24.6	27.9	29.0
4-weeks	Control	23.1	25.8	27.0	28.9
	Intervention	24.3	25.1	27.2	29.0
12-weeks	Control	24.4	26.4	26.8	29.5
	Intervention	24.4	24.5	27.5	29.2
24-weeks	Control	23.4	26.5	27.1	30.1
	Intervention	24.7	24.6	28.3	29.4

3. Supplementary table 3: Mid-calf Indurometer score, mean (SD).

**Time**		**Stage 0**	**Stage P**	**Moderate**	**Severe**
Baseline	Control	3.21 (0.69)	3.23 (0.54)	3.16 (0.55)	3.21 (0.61)
	Intervention	3.49 (0.91)	2.73 (0.83)	3.16 (0.80)	2.91 (0.66)[1]
4-weeks	Control	2.78 (1.10)	2.97 (0.32)	2.81 (0.48)	2.94 (0.58)
	Intervention	2.26 (0.24)	2.61 (0.66)	2.81 (0.65)	2.58 (0.67)[2]
12-weeks	Control	2.54 (0.69)	2.76 (0.32)	2.43 (0.62)	2.37 (0.59)
	Intervention	2.48 (0.73)	2.62 (0.59)	2.75 (0.85)[3]	2.76 (0.83)[4]
24-weeks	Control	2.34 (0.60)	2.54 (0.65)	2.59 (0.66)	2.40 (0.73)
	Intervention	2.35 (0.57)	2.47 (0.67)	2.52 (0.60)	2.42 (0.56)

Between group comparison – 1. p = 0.014, 2. p = 0.008, 3. P = 0.053, 4. p = 0.023

p-values are calculated using two sample t-test.

4. Supplementary Table 4: Frequency and duration of acute attacks and working days lost

	Time	Control	Intervention	p =
**Acute attacks previous one-month**				
Frequency = 0 attacks, n = (%)	Baseline	23 (39%)	29 (44%)	0.68
	24-weeks	48 (83%)	52 (95%)	0.088
Frequency, median (IQR)	Baseline	1 (0, 1)	1 (0, 1)	0.48
	24-weeks	0 (0, 0)	0 (0, 0)	0.056
Frequency, mean (SD)	Baseline	0.78 (0.74)	0.70 (0.74)	0.54
	24-weeks	0.19 (0.44)	0.22 (1.36)	0.88
Duration, median days (IQR)	Baseline	3 (0, 3)	3 (0, 3)	0.36
	24-weeks *	0 (0, 0)	0 (0, 0)	0.046
Duration, mean days (SD)	Baseline	2.64 (3.02)	2.18 (2.60)	0.36
	24-weeks	.67 (1.60)	0.2 (1.01)	0.065
**Acute attacks previous 6-months**				
Frequency = 0 attacks, n = (%)	Baseline	7 (13%)	4 (8%)	0.22
	24-weeks *	36 (62%)	49 (88%)	0.014
Frequency, median (IQR)	Baseline	3 (1, 5)	3 (1, 4)	0.74
	24-weeks *	0 (0, 1)	0 (0, 0)	0.002
Frequency, mean (SD)	Baseline	3.43(4.85)	3.2 (2.71)	0.76
	24-weeks	0.79 (1.55)	0.38 (1.48)	0.14
Duration, median days (IQR)	Baseline *	4 (3, 5)	3 (3, 4)	0.023
	24-weeks *	0 (0, 3)	0 (0, 0)	0.001
Duration, mean days (SD)	Baseline *	5.72 (6.78)	3.45 (1.60)	0.024
	24-weeks *	1.57 (2.56)	0.45 (1.43)	0.005
**Lost working days previous one-month**				
Days = 0 days, n = (%)	Baseline	21 (36%)	32 (48%)	0.34
	24-weeks	48 (83%)	52 (95%)	0.13
Days, median (IQR)	Baseline	3 (0, 6)	1.5 (0, 4)	0.22
	24-weeks	0 (0, 0)	0 (0, 0)	0.057
Days, mean (SD)	Baseline	3.46 (4.04)	3 (4.9)	0.57
	24-weeks	0.78 (1.85)	0.71 (4.14)	0.91
**Lost working days previous 6-months**				
Frequency = 0 days, n = (%)	Baseline	10 (17%)	17 (25%)	0.57
	24-weeks	36 (62%)	47 (84%)	0.24
Frequency, median (IQR)	Baseline	10 (4, 20)	7 (0, 15)	0.17
	24-weeks*	0 (0, 5)	0 (0, 0)	0.009
Frequency, mean (SD)	Baseline	13.58 (13.47)	12.12 (22.72)	0.67
	24-weeks	3.47 (6.13)	1.95 (8.26)	0.27

** Significant between group differences*
